# Relation between Isometric Neck Strength and White Matter Organization in Collegiate Athletes

**DOI:** 10.1089/neur.2020.0025

**Published:** 2020-11-30

**Authors:** Nicola L. de Souza, Emily L. Dennis, Allison M. Brown, Sasha Singh, Elisabeth A. Wilde, Jennifer F. Buckman, Carrie Esopenko

**Affiliations:** ^1^ School of Graduate Studies, Biomedical Sciences, Rutgers Biomedical and Health Sciences, Newark, New Jersey, USA.; ^2^ Department of Neurology, University of Utah School of Medicine, Salt Lake City, Utah, USA.; ^3^ George E. Wahlen Veterans Affairs Medical Center, Salt Lake City, Utah, USA.; ^4^ Department of Rehabilitation and Movement Sciences, School of Health Professions, Rutgers Biomedical and Health Sciences, Newark, New Jersey, USA.; ^5^ Department of Kinesiology and Health, Rutgers University – New Brunswick, Piscataway, New Jersey, USA.

**Keywords:** cervical spine, diffusion tensor imaging, head impacts, soccer

## Abstract

Soccer athletes frequently experience repetitive head impacts (RHI) during games and practices, which may affect neural integrity over time and lead to altered brain structure. Neck strength is hypothesized to limit the transfer of force to the brain and decrease the effect of RHI on brain structure. The goal of our work was to examine whether greater neck strength is associated with more intact white matter organization (WMO) in collegiate athletes exposed to RHI. Collegiate soccer (*n* = 17) and limited/non-contact sport (*n* = 39) athletes were assessed prior to their athletic seasons. Participants completed neck strength assessments using handheld dynamometry in six test positions and diffusion tensor imaging. Fractional anisotropy (FA), mean diffusivity (MD), radial diffusivity (RD), and axial diffusivity (AD) were calculated for 20 white matter (WM) regions. A multi-variate approach was used to examine the relationship between neck strength and diffusion measures in soccer and limited/non-contact athletes. Neck strength was positively associated with FA and negatively associated with RD across several WM regions in soccer players only. Neck strength was not significantly associated with MD or AD in either group. Greater neck strength was related to more intact WMO in athletes with high exposure to RHI, particularly in regions prone to damage from brain trauma such as the basal ganglia, superior longitudinal fasciculus, and frontoparietal WM. Future studies should examine neck strength as a factor to moderate neural outcomes in athletes with exposure to RHI.

## Introduction

Subconcussive impacts occur when force from an impact is transferred to the head, resulting in the brain moving within the skull.^1,2^ Although there are no observable clinical symptoms or a concussion diagnosis at the time of impact, neuronal integrity can be compromised.^1,2^ Further, there is growing concern that repetitive head impacts (RHI) that occur during contact sports may lead to the accumulation of neural damage, contributing to acute alterations in structural integrity^3–5^ and reduced cognitive performance.^6,7^

Soccer is a unique contact sport as players routinely use their head to control and direct the ball. Players head the ball roughly 5–10 times per practice or game,^8,9^ accumulating over 600 impacts per season.^10,11^ Head impacts also result from intentional contact with other players and unintentional impacts with the goal posts or ground.^11^ These impacts appear to contribute to microstructural changes in the brain revealed by advanced neuroimaging techniques.

Diffusion tensor imaging (DTI) measures the diffusion of water molecules within tissues and can detect subtle changes in white matter organization (WMO) better than conventional magnetic resonance imaging (MRI).^12,13^ The magnitude and direction of diffusion can be quantified using: mean diffusivity (MD), the overall level of diffusion; axial diffusivity (AD), diffusion along the longitudinal axis of the axon; radial diffusivity (RD), diffusion perpendicular to the axon; and fractional anisotropy (FA), the directional preference of diffusion ranging from 0 (completely isotropic) to 1 (completely anisotropic).^13,14^ These metrics are proxy measures of white matter (WM) microstructure with each metric representing a different aspect of WM architecture. Thus, inclusion of these four metrics allows for a more nuanced understanding of WMO. Specifically, decreased FA suggests overall WM microstructure damage, whereas decreased AD indicates axonal degeneration. Alternatively, increased MD suggests compromised neural integrity, whereas increased RD indicates myelin damage.^14^

Evidence of WM alterations have been noted in soccer players including reduced FA related to greater heading frequency,^15,16^ decreased AD after one athletic season,^17^ and increased RD relative to non-contact athletes.^18^ Decreased MD^17^ and increased AD,^18^ indicating neuroinflammation associated with RHI, have also been reported. Further, these neural effects seem to compound over time, with evidence of cortical thinning^19^ and altered neurochemistry^20^ associated with chronic RHI exposure in former professional and amateur soccer players. As such, there is a necessity to identify factors that can reduce the effects of RHI on the brain.

Neck strength has been proposed to mediate the magnitude of force transferred to the head upon impact.^21–23^ Specifically, greater neck strength has been associated with reduced linear and rotational head accelerations in collegiate and high school soccer players during heading maneuvers^24,25^ and in other contact sport athletes.^26^ Muscles involved in flexion, extension, rotation, lateral flexion, and flexion in rotation have specifically been shown to reduce acceleration.^21,24–26^ However, there are no studies examining the association of neck strength and WMO in soccer athletes who experience RHI.

The aim of the current study was to examine the relation between neck strength and WMO in collegiate soccer players compared with athletes participating in limited/non-contact sports with limited to no exposure to head impacts. Participants completed isometric neck strength measures and DTI scans. We predicted that greater neck strength would be positively associated with FA and AD and negatively associated with MD and RD in soccer players but not limited/non-contact athletes.

## Methods

### Participants

National Collegiate Athletic Association (NCAA) Division 3 athletes from a northeastern university (*n* = 78) completed neck strength assessments and DTI within a month of the start of their athletic season as part of an ongoing longitudinal study. Athletes completing their pre-season physicals were contacted prior to the start of their athletic season. Fourteen participants were excluded due to concussion history, 6 due to scanner artifacts, and 2 due to being outliers on neck strength measures (>3 standard deviations from the overall group mean). The final sample of 56 athletes was categorized as: contact athletes or soccer players (*n* = 17) who tend to experience high exposure to RHI due to the nature of the sport, or a comparison group of limited/non-contact sport athletes (*n* = 39; e.g., tennis, basketball, volleyball, and cross country running) who experience low to no exposure to RHI. Participant demographics are reported in Table 1. The university's institutional review board approved study procedures and participants provided written informed consent prior to study completion.

**Table 1.** Descriptive Statistics for Demographics and Neck Strength

	All	Soccer	Limited/Non-contact
*N*	56	17	39
Age	19.2 (1.1)	19.0 (1.4)	19.3 (1.0)
Female	28	9	19
Right-handed	49	16	33
BMI^a^	25.1 (3.2)	23.6 (2.6)	25.7 (3.3)
Strength measures			
Extension	12.5 (4.4)	11.3 (3.4)	13.0 (4.7)
Flexion	12.7 (5.3)	11.3 (4.0)	13.3 (5.7)
Right rotation	8.7 (2.1)	8.7 (1.6)	8.7 (2.3)
Left rotation	8.4 (2.3)	8.6 (2.0)	8.4 (2.5)
Right SCM	8.1 (2.8)	7.7 (2.5)	8.3 (2.9)
Left SCM	8.0 (2.8)	7.7 (2.1)	8.2 (3.0)

^a^ BMI was missing for one limited/non-contact sport athlete.

Mean peak force is in kilograms with standard deviation in parentheses.

SCM, sternocleidomastoid; BMI, body mass index (kilograms/meter^2^).

### Measures

Isometric neck strength was collected using handheld dynamometry (Hand Held Dynamometer, Lafayette Instrument Company, Lafayette, IL, USA) in six standardized test positions (flexion, extension, right and left rotation, and right and left flexion in rotation).^27^ Flexion in rotation strength testing assesses several muscles but is defined as the standard test position to isolate the sternocleidomastoid (SCM) muscle and will henceforth be referred to as SCM strength. During the assessment, participants were lying down with their torso fixed to the table using a belt to isolate neck movements.^28^ Three trials of each movement were performed, with each trial consisting of a 3-sec isometric hold followed by a 30-sec rest. Peak force was averaged across the trials. Each neck strength assessment was performed by one of two physical therapists.

Participants completed T1-weighted magnetization prepared rapid gradient echo imaging (MPRAGE; 176 slices, voxel size = 1.0 × 1.0 × 1.0 mm, repetition time [TR] = 1900 msec, echo time [TE] = 2.52 msec, and field of view [FOV] of 256 mm) and DTI (64 directions, 60 slices, voxel size = 2.0 × 2.0 × 2.0 mm, TR = 9000 msec, TE = 99 msec, FOV = 220 mm, and b = 1100 sec/mm^2^) scans on a Siemens 3T Trio scanner.

### Image preprocessing and analysis

The Enhancing NeuroImaging Genetics through Meta-Analysis (ENIGMA)-DTI Working Group DTI pre-processing and analysis pipelines were used (http://enigma.ini.usc.edu/protocols/dti-protocols/).^29^ Briefly, pre-processing involved eddy current correction, echo-planar imaging-induced distortion correction, and tensor fitting. Tensors were mapped and projected onto the ENIGMA-DTI template. Individual subject FA maps were aligned to the custom ENIGMA-DTI FA template derived from 400 adult participants scanned across four sites.^30^

FA voxels were then projected onto the ENIGMA-DTI template skeleton creating a unique FA skeleton in the same space for each participant. The same projections were used for the non-FA (MD, AD, and RD) images. Voxels along the individual skeletons were averaged across WM regions of interest (ROIs). A total of 20 bilateral ROIs were delineated based on the JHU WM atlas, an established WM parcellation derived using deterministic tractography.^31^ A whole-brain WM skeleton was defined according to the tract-based spatial statistic (TBSS) methodology,^32^ and ROI-averaged measures of FA, MD, AD, and RD were calculated by averaging over all skeleton voxels encapsulated by an ROI. This ensured that voxels at the periphery of a fiber bundle, where residual registration misalignment is typically maximal, were excluded from the ROI average. In other words, ROI averaging was performed based on the core of each fiber bundle, as defined by the WM skeleton.

The multi-subject JHU WM parcellation atlas^31^ was used to parcellate ROIs from the ENIGMA template in Montreal Neurological Institute (MNI) space, with updated label identification to correct an earlier atlas error.^33^ We focused on WM tracts that are sensitive to head trauma including the corpus callosum,^3,16,18^ corona radiata,^3,18^ internal and external capsule,^3,18^ cingulum,^3,4,16–18^ fornix,^3,4^ uncinate fasciculus,^17^ superior fronto-occipital fasciculus,^34^ superior longitudinal fasciculus,^16,17^ sagittal stratum,^3^ and posterior thalamic radiation.^35^ Twenty ROIs were extracted from the skeletonized images, including 4 midsagittal regions (no lateralized components) and 16 lateralized regions (Supplementary Table S1). The corticospinal tract was not included due to poor reliability.^29,30^ To ensure data quality, we visually inspected vector directions of the FA images before registration and alignment to the ENIGMA template after registration.

### Statistical analysis

Group differences in neck strength and DTI metrics were compared with independent *t* tests, corrected for multiple comparisons (*p* < 0.0025), in SPSS version 25.0. Partial least squares (PLS) was used to assess the relation between the ROI diffusivity measures (FA, MD, RD, and AD) and neck strength for soccer players and limited/non-contact athletes. PLS is a multi-variate technique for quantifying the pattern and strength of the relationship between two sets of variables.^36,37^ It is a data-driven approach that requires no prior assumptions about the relationship between variables. As all comparisons are entered in one step, there is no need to correct for multiple comparisons.^36^ PLS calculates latent variables (LVs) by singular value decomposition to express the largest amount of information common to both sets of variables (i.e., cross-block variance).^38^ Significant LVs show how the pattern across ROIs relates to the neck strength measures. The significance of LVs was computed by 1500 permutation tests where the ROI diffusivity measures were randomly reordered creating a sampling distribution to determine the probability of LVs occurring by chance.^39^ An LV was considered statistically significant when the probability of the singular value was less than 0.05.

Each ROI diffusivity measure is associated with a weight or “salience” on the LV (similar to a factor loading). To determine the stability of each ROI's contribution to the LV, salience-to-standard error, or bootstrap ratios (BSRs), were calculated with bootstrap estimation in which the ROI diffusivity measures and neck strength variables were resampled 500 times with replacement.^40^ BSRs are interpreted similarly to z-scores such that a BSR larger than 2 corresponds to a 95% confidence limit (*p* < 0.05) and is considered stable. Correlation profiles were also generated to show how the neck strength measures relate to the LV for each group and were computed with 95% confidence intervals that are interpreted as significant when they do not cross zero. A positive association between ROI diffusivity metrics and neck strength measures is indicated when the correlations and BSRs are in the same direction, whereas a negative association is indicated when the correlations and BSRs are in the opposite direction. PLS was performed with a PLS software package^36^ run in MATLAB (R2018a, The Mathworks Inc.). Separate analyses were conducted for FA, MD, RD, and AD.

We also performed an exploratory analysis to examine how the relationship between neck strength and WMO differs based on sex. The multi-variate scores from the significant LVs in the PLS analyses were extracted and plotted. The scores were computed from the PLS analysis where the original matrix was projected onto the saliences from the singular value decomposition.^38^ This produced a pair of scores for each participant, with a neck score characterizing a profile across all neck strength measures and a brain score characterizing the ROI diffusivity metrics across all WM tracts. Interpreting the scores together reflects the relationship between neck strength and WM for each participant.

## Results

Neck strength measures (*p*s > 0.17; Cohen's *d*s < 0.41) and DTI metrics (*p*s > 0.007) did not significantly differ between soccer players and limited/non-contact athletes. The majority of group differences in DTI metrics had negligible to small effect sizes. There were some medium effects: the right external capsule, fornix, and right superior corona radiata for FA and RD; the right cingulate gyrus for FA and AD; the right fornix/stria terminalis for MD and AD; the right anterior limb of the internal capsule and left cingulate gyrus for MD; and the left posterior thalamic radiation and left sagittal stratum for AD. There was also one large effect for the left superior front-occipital fasciculus for AD.

**FIG. 1.**
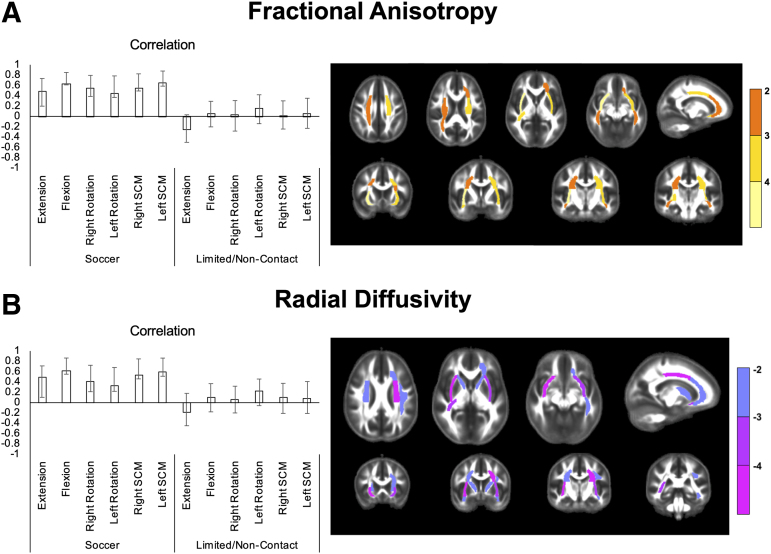
**(A)** A significant LV from the PLS analysis of FA and neck strength indicated a significant positive relation between neck strength and FA in soccer athletes only. **(B)** A significant LV from the PLS analysis examining RD and neck strength revealed a significant negative relation between neck strength and RD in soccer athletes only. *Left panel*: neck muscle strength correlations for flexion, extension, right and left rotation, and right and left SCM. Error bars represent 95% confidence intervals*. Right panel*: BSRs (analogous to a z-score) for WM regions; a BSR threshold of ±2 corresponds to a *p*-value of *p* < 0.05. Warm colors indicate a positive correlation with neck strength; cool colors indicate a negative correlation. BSRs, bootstrap ratios; FA, fractional anisotropy; LV, latent variable; PLS, partial least squares; RD, radial diffusivity; SCM, sternocleidomastoid; WM, white matter.

The PLS analysis examining the relation between FA and neck strength in soccer players and limited/non-contact athletes indicated one significant LV (*p* = 0.045, 70.9% of cross-block variance). Greater neck strength across all positions tested was associated with higher FA in the left anterior corona radiata, bilateral external capsule, right uncinate fasciculus, right posterior corona radiata, right retrolenticular part of the internal capsule, bilateral superior corona radiata, and bilateral sagittal stratum in soccer players only (Fig. 1A). The analysis examining the relationship between RD and neck strength also identified one significant LV (*p* = 0.043, 77.8% of cross-block variance). Greater neck strength across all positions tested was associated with lower RD in the left anterior corona radiata, bilateral anterior limb of the internal capsule, bilateral external capsule, bilateral uncinate fasciculus, right retrolenticular part of the internal capsule, bilateral superior corona radiata, left superior longitudinal fasciculus, and left sagittal stratum in soccer players only (Fig. 1B). The analysis examining the relationship between MD and neck strength revealed a similar pattern, but the LV was not significant (*p* = 0.083). No significant patterns were found between AD and neck strength.

**FIG. 2.**
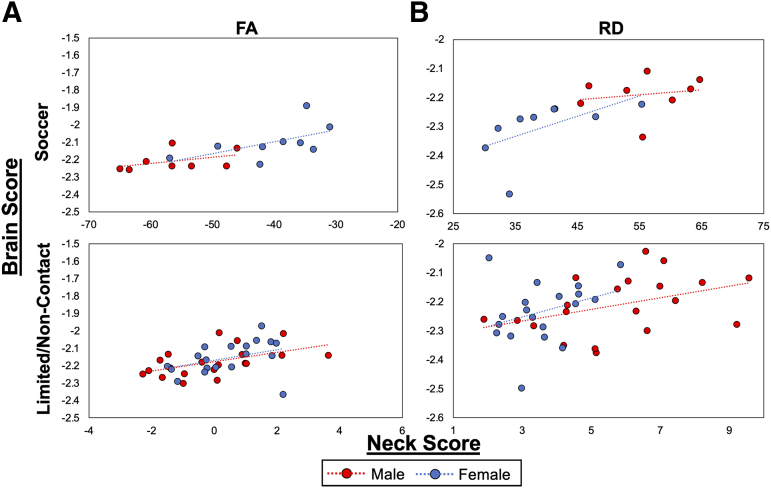
Multi-variate neck scores plotted by brain scores from the significant LVs from the PLS analyses of **(A)** FA and **(B)** RD and neck strength in soccer and limited/non-contact athletes. Each point reflects the relationship between neck strength and WM for each participant. Red circles = male athletes; blue circles = female athletes. FA, fractional anisotropy; LVs, latent variables; PLS, partial least squares; RD, radial diffusivity; WM, white matter.

To assess the influence of sex on the relation between neck strength and WMO, the multi-variate neck and brain scores from the FA and RD PLS analyses were plotted for contact and non-contact athletes (Fig. 2).The plots demonstrated different patterns dependent on sex for soccer athletes, where female soccer players tend to have a stronger relationship between neck strength and WMO. For limited/non-contact athletes, the plots showed weak or no relationship between neck strength and WMO that did not differ between males and females.

## Discussion

RHI have been linked to acute^15–18^ and chronic^19,20^ neural changes. The frequent use of the head to direct the ball and other intentional and unintentional impacts characteristic of soccer participation increase soccer athletes' risk for detrimental acute and chronic neural changes. Neck strength may reduce the effect of head impacts on the brain as stronger neck muscles can stabilize the head and reduce the transfer of energy to the brain upon impact.^22,23^ Athletes with weaker necks experience greater peak acceleration and displacement upon impact.^26,41^ Greater neck strength also seems to translate to a reduction in injury risk: for every 1-lb increase in neck strength, there is a 5% reduction in the odds of sustaining a concussion in high school athletes.^42^ Further, athletes in the year following concussion show reduced neuromuscular control and thus experience higher linear accelerations, potentially increasing susceptibility to damage from RHI.^43^ However, the neural mechanisms underlying the protective role of neck strength have not been assessed. This preliminary study, to our knowledge, is the first to characterize the relationship between neck strength and WMO in athletes who experience RHI. We found that greater neck strength was associated with more intact WMO (i.e., higher FA and lower RD) in several ROIs of collegiate soccer players at the start of their athletic season, but not limited/non-contact athletes. These findings lay the groundwork for future studies to examine whether stronger neck muscles may be protective against changes in WMO due to exposure to RHI.

### Stronger necks may protect against reductions in WMO

Although not all impacts will result in concussion, over time the linear and rotational head accelerations due to RHI may result in damaged WM microstructure. Over one competitive season, altered WM has been correlated with the number of head impacts with high rotational and linear acceleration.^44^ In addition, specific WM tracts are expected to be affected by these RHI. The “cone of vulnerability” describes brain regions that are most commonly damaged after sudden acceleration/deceleration deformation of the brain, including the basal ganglia, superior longitudinal fasciculus, and frontoparietal WM^34^—regions found to be associated with neck strength in this study. Thus, our results are consistent with the theory that greater neck strength better stabilizes the head during impact, minimizing movement of the brain resulting in reduced shearing of axons.^22,23^ Specifically, elevated RD and reduced FA may indicate myelin damage that is observed as greater diffusion orthogonal to the axon^45,46^ with a corresponding loss of proportional diffusion along the main axis of the axon.^14,47^ Therefore, soccer players with stronger necks may be more resistant to disrupted axonal architecture and demyelination that occurs due to RHI. Further, the association of neck strength and more intact WMO was found in WM tracts within the frontal region, and likely one location of impact, such as the anterior and superior corona radiata and uncincate fasciculus, and within tracts (e.g., superior longitudinal fasciculus) that would be stretched as the brain accelerates after contact.^34^

Contrary to expectation, however, we did not find significant associations between neck strength and MD or AD. Although we had predicted greater neck strength would be associated with lower MD, the pattern between neck strength and MD was not significant (*p* = 0.083). We had also predicted that AD would be positively associated with neck strength, as lower AD is thought to reflect axonal damage.^46^ The lack of association may suggest that different tissues have varying thresholds to relatively mild physical strain (i.e., compared with a sports-related concussion [SRC] or moderate/severe traumatic brain injury) or different speeds of recovery.^47^

Female athletes consistently show lower neck strength relative to male athletes, which may be one factor underlying the increased linear and rotational head acceleration experienced by female athletes due to head impacts, as noted in the literature.^24,41,48^ As such, exploratory analyses assessing sex differences in the association between neck strength and WMO for soccer and limited/non-contact sports were used. We found that female soccer players had a stronger relationship between neck strength and WMO relative to male soccer players. There were no such sex differences in the data for limited/non-contact athletes. This may provide one mechanism underlying studies showing that female athletes tend to show more WM alterations after exposure to RHI^49^ and worse outcomes after concussion compared with male athletes.^50^ However, these results are preliminary and need to be considered in light of the limited samples of male and female soccer players; the relationship should be explored in a larger, sex-matched sample to determine how sex differences in neck strength may affect brain structure in athletes exposed to RHI.

Together, our results coupled with past work showing that greater neck strength reduces head accelerations,^21,24,25^ suggest that players with weaker necks may experience greater linear and rotational head acceleration upon impact resulting in greater movement of the brain and more extensive structural damage. Our exploratory analyses also suggest that these patterns may be affected by sex, but this needs to be confirmed in larger studies. Still, the findings of this study have important implications given previous research linking RHI-associated WM changes to cognitive performance.^15,51^ Specifically, a recent article showed that WMO is associated with cognitive function in amateur soccer players where greater volume of low diffusivity (RD, MD, and AD) was associated with better verbal memory, processing speed, and attention.^51^ Moreover, athletes with low or no heading exposure showed greater expression of low RD and high FA relative to players with high heading exposure. Future research should examine the relationship between neck strength, WMO, and cognitive performance.

### Limitations and future directions

Our preliminary findings identify a plausible mechanism for the protective effect that neck strength may have in athletes with exposure to RHI, but there are some limitations that must be considered. The study used a cross-sectional design and therefore WM alterations cannot be interpreted in terms of within-subject changes. Moreover, athletes were examined once within a month of the beginning of their season and thus we could not address whether alterations in WMO were influenced by cumulative exposure to RHI within and across competitive seasons and over a collegiate athletic career. Another limitation was the small sample of soccer athletes. Further, the samples for soccer and limited/non-contact athlete groups were not matched and the study was not powered to examine how other variables, such as sex, influence the relation between neck strength and WMO. This is an important future direction to consider as female compared with male athletes show lower neck strength and greater linear and rotational head acceleration upon impact.^24,41,48^ Finally, we could not determine how RHI exposure history was related to WMO. This study did not collect RHI exposure history nor quantify the number and force of impacts experienced; future studies should assess whether these factors mediate the relationship between neck strength and WMO. Prospective studies are also needed to examine whether greater neck strength attenuates damage to WMO following a diagnosed SRC.

## Conclusion

We assessed the relation between neck strength and WMO in collegiate athletes with high and low exposure to RHI. We found that soccer players with stronger necks had more intact WMO, suggesting that neck strength may have a role in reducing the WM alterations associated with RHI. Therefore, neck strength is a promising target for further investigation as a modifiable factor to reduce the risk of brain trauma and associated cognitive effects in athletes with high exposure to RHI.

## Supplementary Material

Supplemental data

## Abbreviations Used

AD: axial diffusivity
BSR: bootstrap ratio
DTI: diffusion tensor imaging
ENIGMA: Enhancing NeuroImaging Genetics through Meta-Analysis
FA: fractional anisotropy
FOV: field of view
LV: latent variable
MD: mean diffusivity
MNI: Montreal Neurological Institute
MPRAGE: magnetization prepared rapid gradient echo imaging
MRI: magnetic resonance imaging
NCAA: National Collegiate Athletic Association
PLS: partial least squares
RD: radial diffusivity
RHI: repetitive head impacts
ROI: region of interest
SCM: sternocleidomastoid
SRC: sports-related concussion
TBSS: tract-based spatial statistic
TE: echo time
TR: repetition time
WM: white matter
WMO: white matter organization
