# Supplementary material for: Relation between Isometric Neck Strength and White Matter Organization in Collegiate Athletes
Source: Neurotrauma Rep. 2020 Nov 30;1(1):232–40. doi: 10.1089/neur.2020.0025 (PMC8240886; doi:10.1089/neur.2020.0025)
Supplement: Supplemental data [file Supp_TableS1.docx]

**Supplemental Table 1.**

White matter regions of interest. Twenty regions were extracted from the skeletonized images, including 4 midsagittal regions (no lateralized components), and 16 lateralized regions (left [L] and right [R])

| **Abbreviation** | **Tract Name** |
| --- | --- |
| ACR (L+R) | Anterior corona radiata |
| ALIC (L+R) | Anterior limb of internal capsule |
| SCC | Splenium of corpus callosum |
| BCC | Body of corpus callosum |
| GCC | Genu of corpus callosum |
| CGC (L+R) | Cingulum (cingulate gyrus) |
| CGH (L+R) | Cingulum (hippocampus) |
| EC (L+R) | External capsule |
| FX | Fornix |
| FX/ST (L+R) | Fornix (cres) / Stria terminalis |
| UNC (L+R) | Uncinate fasciculus |
| PCR (L+R) | Posterior corona radiata |
| PLIC (L+R) | Posterior limb of internal capsule |
| PTR (L+R) | Posterior thalamic radiation |
| RLIC (L+R) | Retrolenticular part of internal capsule |
| SCR (L+R) | Superior corona radiata |
| SFO (L+R) | Superior fronto-occipital fasciculus |
| SLF (L+R) | Superior longitudinal fasciculus |
| SS (L+R) | Sagittal stratum |
| TAP (L+R) | Tapetum |
